# NONO and tumorigenesis: More than splicing

**DOI:** 10.1111/jcmm.15141

**Published:** 2020-03-13

**Authors:** Peifu Feng, Ling Li, Tanggang Deng, Yan Liu, Neng Ling, Siyuan Qiu, Lin Zhang, Bo Peng, Wei Xiong, Lanqin Cao, Lei Zhang, Mao Ye

**Affiliations:** ^1^ Molecular Science and Biomedicine Laboratory State Key Laboratory for Chemo/Biosensing and Chemometrics College of Biology College of Chemistry and Chemical Engineering Collaborative Innovation Center for Molecular Engineering for Theranostics Hunan University Changsha China; ^2^ Ophthalmology and Eye Research Center the Second Xiangya Hospital Central South University Changsha China; ^3^ Department of Gynecology Xiangya Hospital Central South University Changsha China; ^4^ Department of Nephrology the Second Xiangya Hospital Central South University Changsha China

**Keywords:** DBHS, NONO, splicing, tumorigenesis

## Abstract

The non‐POU domain‐containing octamer‐binding protein NONO/p54^nrb^, which belongs to the Drosophila behaviour/human splicing (DBHS) family, is a multifunctional nuclear protein rarely functioning alone. Emerging solid evidences showed that NONO engages in almost every step of gene regulation, including but not limited to mRNA splicing, DNA unwinding, transcriptional regulation, nuclear retention of defective RNA and DNA repair. NONO is involved in many biological processes including cell proliferation, apoptosis, migration and DNA damage repair. Dysregulation of NONO has been found in many types of cancer. In this review, we summarize the current and fast‐growing knowledge about the regulation of NONO, its biological function and implications in tumorigenesis and cancer progression. Overall, significant findings about the roles of NONO have been made, which might make NONO to be a new biomarker or/and a possible therapeutic target for cancers.

## 1 INTRODUCTION

The NONO (non‐POU domain‐containing octamer‐binding protein) protein, also known as 54 kD nuclear RNA‐ and DNA‐binding protein (p54nrb), belongs to the multifunctional DBHS (Drosophila behaviour/human splicing) family of proteins which can bind DNA, RNA and protein.^1^ NONO has a nuclear localization signal (NLS) at its C‐terminal, so it is located in the nucleus of most mammalian cells and is primarily distributed in the subnuclear domain named paraspeckles.^2^ Emerging evidence strongly indicates new roles for NONO in tumorigenesis, including but not limited to regulating proliferation, apoptosis, cell migration and DNA damage repair. Here, we provide a comprehensive review of the NONO and its functions in tumorigenesis.

## 2 STRUCTURE

**Figure 1:**
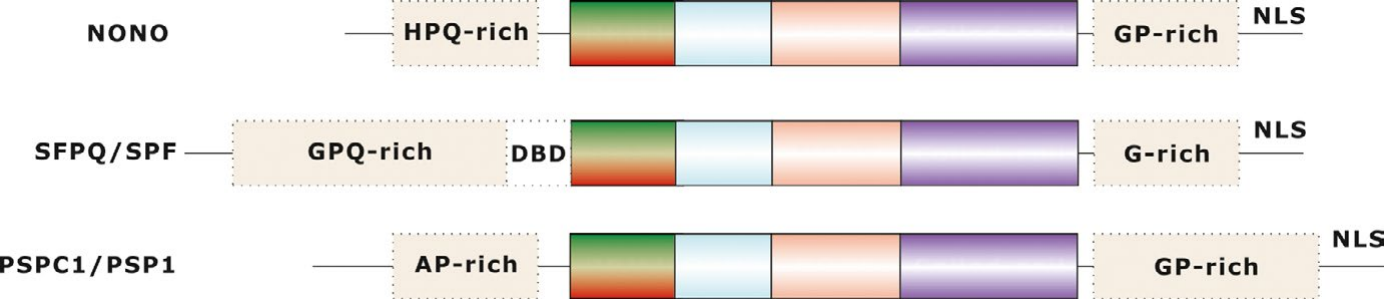
Schematic representation of human DBHS protein domain architecture. The uncharacterized DBD of SFPQ and other low complexity regions of each paralog are indicated in dashed boxes

The human *NONO* gene is located on chromosome X 13p1 and encodes a 471 aa protein identified as a homolog of the Drosophila NONA/BJ6 from Hela cells.^1^ NONO is one of the three homologous mammalian proteins that are termed the ‘DBHS’ family, the others being SFPQ/SPF (splicing factor proline and glutamine rich) and PSPC1/PSP1 (paraspeckle component 1). The DBHS proteins share a core region ‘DBHS’ of ~300 amino acids, which is characterized by highly conserved N‐terminal RNA recognition motifs (RRMs), a NOPS (NONA/paraspeckle domain) and a C‐terminal coiled‐coil and are largely regarded as nuclear factors.^1^, ^3^ RRM domains recognize and interact with RNA and single strand DNA.^4^ The NOPS domain is in charge of mediating DBHS dimerization, and sometimes some surface‐exposed basic residues (R280 and R284) within the NOPS domain may serve as a single molecular interaction site to bind nucleic acids.^5^ The C‐terminal end facilitates dimerization and oligomerization.^6^ However, there are structural differences outside the ‘DBHS region’ between members of the family (Figure 1). Moreover, like other two DBHS proteins, NONO rarely functions alone, its interactions are regulated by its structure changes and largely regulated by post‐translational modifications and the interactors.^6^

## 3 REGULATION OF NONO EXPRESSION

### 3.1 Transcription

NONO is involved in collagen formation and fibrosis in some situations.^7^, ^8^, ^9^, ^10^ In the patients with aortic dissection (AD), there are significant correlations between NONO and collagen. NONO protein is decreased in AD tissue compared with control tissue, its mRNA expression is also decreased.^10^ NONO is also regulated at the transcriptional level in melanoma, because MIA (melanoma inhibitory activity) depletion can reduce significantly NONO mRNA and protein level (see Section 6.5 below).^11^ The detailed mechanism about transcriptional regulation of NONO still needs much more in‐depth studies.

### 3.2 mRNA stability

NONO can regulate the intra‐S‐phase checkpoint in response to UV radiation.^12^ However, UV rays could induce the expression of a microRNA, miR‐320a, which could target NONO mRNA for degradation by binding its 5′‐UTR. Interestingly, the RNA binding protein HUR (also called ELAVL1), which was also induced by UV rays, was shown to protect NONO mRNA from mir‐320a‐mediated degradation by binding an overlapping site within the 5′UTR.^13^ Later, it was shown that UV induce NONO protein degradation mediated by the RNF8 ubiquitin ligase and interfering with this process affects the S phase checkpoint, consistently with previous work.^14^ Further mechanisms of NONO mRNA regulation still need to be defined.

### 3.3 Post‐translational modifications

Structural and biological data suggest DBHS proteins rarely play their biological roles alone, their interactions with various proteins are regulated by post‐translational modifications.^6^ NONO were proved to be phosphorylated in mitosis in some independent studies.^15^, ^16^, ^17^, ^18^, ^19^, ^20^, ^21^, ^22^ CDK1 phosphorylates T412, T430 and T452 in the C‐terminal extremity of NONO, subsequently the prolyl isomerase Pin1 interacts with the phosphorylated NONO. Furthermore, Pin1 interaction with NONO depends on multisite phosphorylation.^15^ CDK1 also can phosphorylate T15 in the N‐terminal of NONO in vitro. Two independent studies^21^, ^23^ found that NONO could be tyrosine‐phosphorylated; however, they could not exclude that the p‐Tyr antibodies could non‐specifically bind NONO, and a p‐Tyr antibody was found having non‐specific binding affinity to NONO in another study later.^22^ Furthermore, crystal structure of NONO shows that the five Tyr residues of NONO are not in favourable positions to be phosphorylated because of steric hindrance.^4^ Even though, the tyrosine residues still regulate NONO’s multifarious nuclear functions.^22^

CARM1, also known as PRMT4, can methylate NONO, and R357, R365 and R378 are the major sites to be methylated.^24^ CARM1 knock‐down enhances the nuclear retention of mRNAs containing inverted repeated *Alu* elements (IR*Alu*s), via reducing binding of NONO to target mRNAs.^24^ SUMOylation,^25^ ADP‐ribosylation^26^ and acetylation^27^ are also found in several proteomics studies, there should more in‐depth studies.

NONO half‐life is about 32 hours in Hela cell which is consistent with in silico predictions.^28^ Recently, there are some solid evidences proving that NONO turnover can be regulated in vivo.^14^, ^28^ NONO can be polyubiquitinated upon FBW7α^28^ or RNF8^14^ interaction, and three of total 27 lysine residues of NONO are important ubiquitination sites.^14^

## 4 NONO AND GENE REGULATION

**Figure 2:**
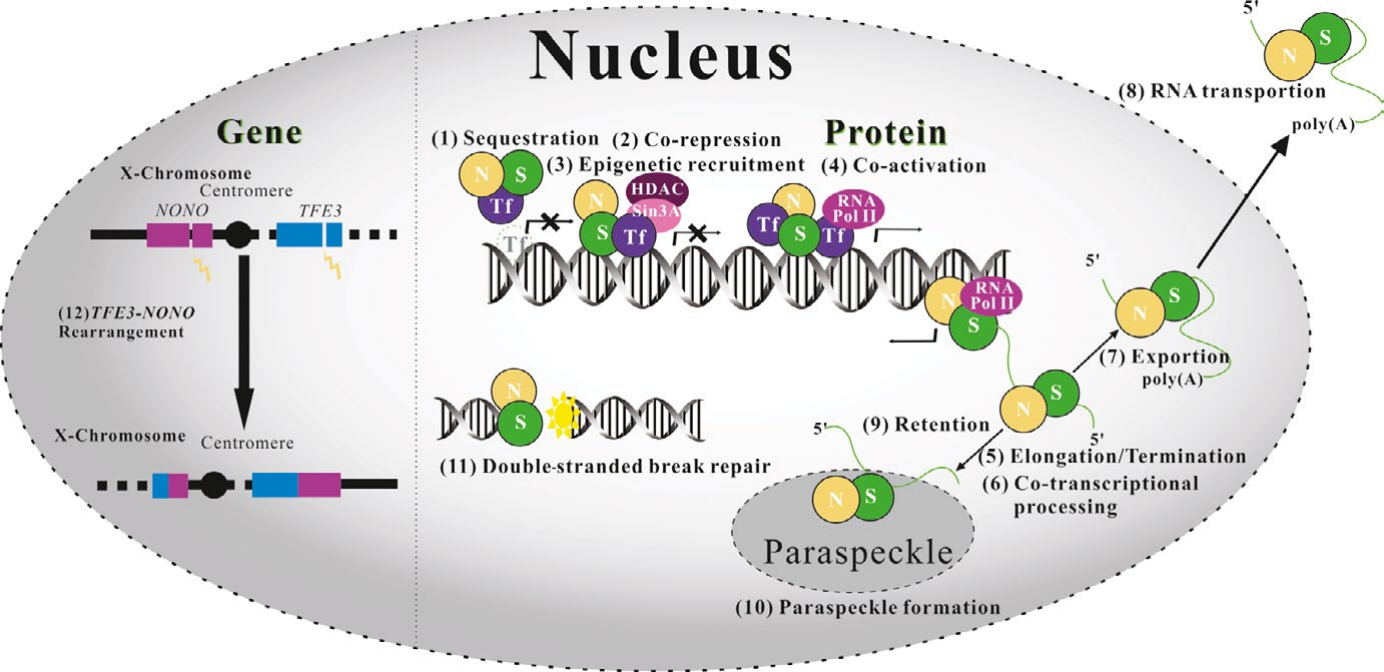
Simplified schematic representation of NONO protein function. The DBHS proteins SFPQ (S) and NONO (N) are represented as simple green and orange spheres respectively. (1) SFPQ and NONO can sequester transcription factors away from target promoters, (2) act as co‐repressors at target promoters and (3) in complex with repressors stimulate epigenetic silencing. (4) Both SFPQ and NONO are associated with co‐activation of transcription through (5) elongation up to termination. (6) SFPQ and NONO also remain associated with nascent mRNA to facilitate co‐transcriptional processing, (7) messenger ribonucleoprotein (mRNP) export and (8) cytosolic trafficking. (9,10) By virtue of their involvement in paraspeckle formation and integrity, SFPQ and NONO can facilitate nuclear RNA retention. (11) SFPQ, NONO and PSPC1 are also involved in double‐stranded break repair. (12) *TFE3‐NONO* rearrangement

NONO engages in almost every step of gene regulation,^29^ including but not limited to pre‐mRNA splicing,^30^, ^31^, ^32^, ^33^ activation of transcription,^32^, ^34^ termination of transcription,^35^ nuclear retention of defective RNA,^36^, ^37^ DNA unwinding,^29^, ^38^ double‐stranded break repair^39^, ^40^ and maintaining correct circadian clock functions^7^, ^41^ (reviewed in Refs. 6, 29) (Figure 2).

NONO can activate the RNA transcription, most of which is nascent RNA. NONO interacts with other promoters of many transcriptionally active genes, such as rhodopsin,^34^ oct4,^42^ TORCs (transducer of regulated CREB)^43^ and AR (androgen receptor),^44^, ^45^ subsequently promotes transcription, which is often associated with a synergistic effect with other promoters.^34^ Sometimes, NONO binds to a suppressor to be prevented from transcription activation, for example, SOCS3 is a suppressor in NONO‐SOCS3 complex, after IL‐1β disrupts the interaction of NONO‐SOCS3, the downstream Mucin8 level increases in transcription level.^46^ Interestingly, on some other contexts, such as DBHS dimer composition, modification status, cell lines, and cellular localization, NONO co‐represses/co‐activates AR‐mediated transcription.^47^, ^48^ NONO can activate basal and cAMP‐dependent transcription of CYP17 gene,^49^, ^50^ Sin3A‐HDAC (histone deacetylases) and the binding of SF‐1(steroidogenic factor‐1)/PSF/NONO to the promoter determine the transcription activity.^50^ Through recruitment of epigenetic regulator HDAC, SFPQ/NONO can act on hormone receptors such as the thyroid and retinoid X receptors.^51^ In some cases, NONO represses transcription by sequestering activators away from target promoters. For instance, SFPQ/NONO prevents the progesterone receptor to bind to PR DNA, subsequently represses the transcription.^52^

mRNA splicing is a critical step in the post‐transcriptional gene regulation and expands functional proteome in eukaryotes. NONO has been identified in splicing‐related complexes in a proteomic study,^31^ it is homologous to another splicing‐related protein SFPQ/PSF and they associate with each other.^29^ NONO is not an essential component in spliceosome assembly or splicing^53^; however, it interacts with other spliceosomes and promotes splicing via the distal 5′ splicing site in pre‐mRNA alternative splicing.^30^, ^53^, ^54^ NONO also additionally interacts with the C‐terminal of RNA pol IIa and IIo.^30^, ^55^ In some conditions, NONO can also interact with 3′ end of mRNA splicing such as TNF‐a,^56^ the exonuclease XRN2,^35^ the snRNP‐free U1A^57^ and GLA (α‐galactosidase A).^58^

When it interacts with the C‐terminal of RNA pol II (RNAPII), NONO not only regulates the mRNA splicing, but also associates with transcriptional elongation and termination.^30^ Sometimes, NONO/SFPQ interacts with C‐terminal of both phosphorylated and unphosphorylated RNAPII and nascent RNA in the same time, because differently phosphorylated forms of RNAPII associate with the dynamic mRNA processing.^59^

NONO is also snRNA export stimulatory factor, accelerating the recruitment of PHAX for efficient nuclear export of snRNA, NONO and PSF form a heterodimer in this step.^37^

## 5 PHYSIOLOGICAL FUNCTIONS OF NONO

### 5.1 Cell proliferation

To date, it is known that NONO can induce/promote cell proliferation in a wide variety of cell types including tumour cells. In Hela,^12^ MCF‐7^60^ and Mel Im^11^ cells, NONO silencing reduces the cell proliferation rate, and NONO‐silenced cells have a delayed G1/S transition,^12^ whereas the two other DBHS proteins have not significant effects on the growth of MCF‐7 cells.^60^ Further studies demonstrated that NONO promotes breast cancer cell proliferation through sterol regulatory element‐binding protein a (SREBP‐a).^60^ SREBPs are transcription factors that bind to the sterol regulatory element DNA sequence TCACNCCAC and activate the transcription of genes associated with the biosynthesis of fatty acids and cholesterol.^61^, ^62^ NONO regulates SREBP‐1a protein levels in the nucleus through a post‐transcriptional mechanism.^60^ Erk1/2/MAPK and PI3K/AKT activation are frequent events in oesophageal squamous cell carcinoma (ESCC), and the expression levels of phosphorylated (activated state) Erk1/2 and AKT are dramatically decreased in NONO knock‐down cells.^63^ Taken together, the Erk1/2 and PI3K/AKT pathways may be required for the NONO‐regulated growth of ESCC cells,^63^ unfortunately the exact mechanism is still not elucidated.

NONO also can inhibit cell proliferation in certain conditions. NONO was proved as a transcriptional activator of p16‐INK4A, an important checkpoint gene‐associated cell cycle. The fibroblast deficient of NONO shows increased proliferation due to low levels of p16‐INK4A. In some breast cancers, lower NONO is associated with increased proliferation.^64^, ^65^ The THP1 cell knock downed NONO shows a decrease of G0/G1 phase cells and an increase of S and G2/M phase cells compared with wild‐type or negative control cells, which indicates the knock‐down of NONO can accelerate THP1 cell cycle.^66^ Thus, NONO plays dual roles as either a promoter or inhibitor of cell proliferation.

### 5.2 Apoptosis

Similar to its effect on proliferation, NONO plays a dual effect on apoptosis. In ESCC,^63^ melanoma^11^ and NONO^gt^ mice cells, NONO knock‐down/deficiency significantly increases cell apoptosis, including early apoptosis and late apoptosis; moreover, the apoptosis was mediated by the activation of caspase‐3 and then led to PARP binding to target DNA.^63^ Polypyrimidine tract‐binding protein (PTB) is critical in apoptosis.^67^ Remodelling of a PTB‐containing complex occurs following treatment to induce apoptosis, meanwhile, the IRES (Internal Ribosome Entry Segment)‐inhibitory protein hnRNPA1 decreases in association with PTB. NONO interferes with the association between hnRNPA1 and PTB, then stabilize hnRNPA1 resulting acceleration of apoptosis rates via changing gene expression at post‐transcriptional level.^67^

### 5.3 DNA damage repair

Homologous recombination (HR) and non‐homologous end joining (NHEJ) are two primary double‐strand break (DSB) repair pathways. The NONO/PSF complex is a principal candidate and component of the end‐joining stimulatory fraction that cooperates with other proteins known to participate in NHEJ in vivo,^68^ NONO knock‐down delays NHEJ kinetics in vitro.^7^, ^69^ Although NONO knock‐down has no effects on long‐term survival of cells, attenuated NONO expression can sensitize cells to ionizing irradiation, suggesting that NONO is crucial for DNA DSB repair.^70^ NONO knock‐down delays the resolution of γH2AX foci, increases chromosomal aberrations at the first metaphase following radiation exposure, impairs the recovery from DNA damage,^39^ and decreases clonogenic survival in vivo.^40^ Proteins involved in DSB repair via NHEJ co‐immunoprecipitate with NONO, and rapid recruitment of SFPQ·NONO to DNA damage sites are found after U2OS cells are induced by a laser microbeam,^39^ suggesting that the SFPQ·NONO complex is involved in the early stages of the DSB response.^39^ NONO is also a PAR (poly(ADP‐ribose))‐binding protein and its recruitment to DNA damage sites is PAR‐dependent.^70^ In the other stable reporter cell line, which can monitor HR repair pathway, knock‐down of NONO shows up‐regulation of HR by ~40%. Taken together, NONO not only facilitates NHEJ but also represses the other major DSB repair pathway (HR). Interestingly, NONO promotes NHEJ and represses HR in vivo in the same pathway as PARP‐1.^70^

### 5.4 Cell migration

NONO is strongly expressed in melanoma,^11^ mPCa (metastatic prostate cancer)^71^ and ESCC,^63^ and its knock‐down significantly reduces cell migration. NONO knock‐down enhances cells attachment to laminin, poly‐l‐lysine^11^ and the surface of culture plates,^63^ but interestingly, it has no effects with regard to fibronectin. These results suggest that NONO influences the ability to attach to components of the extracellular matrix.

A large number of lncRNAs are pervasively transcribed from the human genome, and aberrant expression of lncRNAs may cause abnormal cell functions, leading to various pathological conditions including cancer metastasis.^72^ The increased expression of GAPLINC (gastric adenocarcinoma predictive long intergenic noncoding) RNA was found to be positively correlated high metastasis, NONO protein bound to GAPLINC and reversed the cell invasion effects.^73^ Another lncRNA, MetaLnc9 is correlated with cell migration, NONO interacts with MetaLnc9 and reinforces a positive feedback loop for metastasis as a coactivator for the transcription factor CREB (cAMP response element‐binding protein).^74^ Nevertheless, the detailed molecular mechanism is still not elucidated.

## 6 NONO AND CANCERS

To date, all DBHS proteins have been found to be associated with cancers as either oncogenes or tumour suppressors, including NONO.^6^ Emerging evidence has demonstrated that NONO is overexpressed in various kinds of cancers, including bladder cancer,^75^ lung cancer,^74^, ^76^ prostate cancer^77^, ^78^ and ESCC.^63^ Furthermore, NONO protein level is an independent prognostic factor for some cancers.^71^, ^79^, ^80^, ^81^ By contrast, NONO is usually down‐regulated in ER (oestrogen receptor)‐negative breast cancer. At the gene level, renal cell carcinoma (RCC) is associated with *TFE3* (transcription factor E3)‐*NONO* fusion.^82^

### 6.1 Chromosomal translocation/fusion involving with NONO gene and papillary renal cell carcinoma

Renal cell carcinoma is the most common cancer of the kidney. Based on the histological appearance, RCC can be divided into several subsets including papillary renal cell carcinoma which is characterized by the expression of TFE3 fusion proteins. The *TFE3‐NONO* gene fusion was described for the first time in 1997^83^ (Figure 2:12). The X chromosome inversion inv(X) (p11.2; q12), which results in the fusion of the *NONO* gene and *TFE3*, is a cytogenetically defined translocation. In 2016, the *TFE3‐NONO* RCC morphology was described for the first time, showing subnuclear vacuoles that lead to frequent distinctive suprabasal nuclear palisading.^84^ Furthermore, fusion of *TFE3* and *NONO* is associated with the loss of normal *TFE3/NONO* transcripts.

### 6.2 The CREB‐NONO axis in lung cancer

The CREB has two coactivator families, CBP/p300 and TORCs. The cAMP signal‐transduction pathway can activate transcription by stimulating interactions between CREB, CBP/p300 and TORCs.^85^, ^86^, ^87^ NONO is a TORC2 interactor and can act as a bridge between the CREB/TORC complex and RNA polymerase II to regulate cAMP‐mediated transcription. In A549 cells, the interaction of Gal4‐NONO and CRTC (CREB‐regulated transcription coactivator) is reduced after depletion of LINC00473, which encodes an intergenic lncRNA from the chromosome 6q27 locus. In contrast, LINC00473 overexpression promotes NONO‐CRTC interaction, suggesting that LINC00473 facilitates the interaction of NONO and CRTC and subsequently promotes the cAMP‐mediated transcription of various genes.^76^ Lung cancer patients with high LINC00473 expression had more aggressive pathological behaviours and shorter survival times by enhancing the interaction between NONO and CRTC.^76^ Another lncRNA, MetaLnc9, is overexpressed in non‐small cell lung cancer (NSCLC), subsequently causing poor prognosis and enhanced metastasis formation in patients with NSCLC. Like LINC00473, MetaLnc9 also interacts with NONO to promote the CRTC‐mediated transcription of CREB that offers a positive feedback loop for metastasis.^74^

### 6.3 Breast cancer and NONO: the dual function

Tumorigenesis is a multi‐step process from normal cells to malignancy.^88^ Some proteins, such as p53^89^ and p21,^90^ have dual‐ or multi‐function in a context‐dependent manner. Likewise, NONO was identified as a cancer promoter or suppressor depending on the breast cancer subtype. There are statistically significant association results between NONO expression, tumour hormonal phenotype and mean tumour size.^65^ In contrast to ER + human breast tumours, NONO protein expression was decreased in ER‐ breast cancers.^64^ Moreover, NONO expression in ER‐ breast cancers or NONO variants in ER + cancers might inform on breast tumour progression.^64^

### 6.4 NONO endowing prostate cancer with drug resistance

PCa is one of the most commonly diagnosed cancers in men worldwide and a leading cause of cancer‐related death.^91^ NONO is usually overexpressed in human prostate cancer,^78^, ^92^ and pathological results demonstrated that higher NONO expression correlated with poor prognosis. The relationship between NONO and AR shows a positive correlation too,^92^ and NONO knock‐down can effectively reduce the expression of AR/AR‐V7 at the mRNA and protein levels.^71^ Hormone therapy is an important method to treat prostate cancers,^93^, ^94^ and androgen deprivation therapy (ADT) is the main treatment for aggressive PCa.^95^ Unfortunately, like many other cancer types, resistance is a frequent event associated with PCa, such as castration‐resistant prostate cancer (CRPC).^96^, ^97^ Prostate cancer gene expression marker 1, also called PCGEM1, is a lncRNA which is often up‐regulated in prostate cancers and has been implicated in resistance to anticancer drug‐induced apoptosis.^98^ In CRPCs, NONO induces PCGEM1 expression and subsequently up‐regulates AR level, which promotes castration‐resistance in PCa.^71^, ^77^, ^78^

### 6.5 NONO regulating the progression of melanoma

MIA, which is secreted by melanoma cells, has been used as a tumour marker. Increased MIA serum level is related to metastatic disease or tumour recurrence^99^; it also likely represents a key molecule that regulates melanoma progression.^100^ NONO is a downstream target of MIA, and MIA knock‐down reduces NONO expression at both the mRNA and protein levels. NONO has its own downstream targets, such as Cx‐43, which allows for intercellular gap junction communication between cells to regulate cell death, proliferation and differentiation.^101^ NONO is highly expressed in malignant melanoma compared with melanocytes, which subsequently inhibits Cx‐43 expression. NONO not only plays an important role in the early steps of tumour formation and in the anti‐apoptosis process but also influences the migratory potential of melanoma cells; therefore, NONO may be involved in the MIA‐mediated metastasis of melanoma cells.^11^ Exposure to ultraviolet (UV) radiation, namely UVA (315‐400 nm) and UVB (280‐315 nm), is a major risk factor for melanoma development, as it can cause direct DNA damage.^102^ The NONO/PSF complex is identified as a stimulatory fraction repair system in mammalian cells that promotes DSB repair, thus confers increased radio‐sensitivity to cells,^40^ while NONO silencing affects the UVC‐induced DNA damage response in melanoma cells.^12^ Thus, its involvement in the rapid and accurate repair of DSBs makes NONO an efficient target of radiosensitizers.

### 6.6 Others

Colorectal cancer is one of the most commonly occurring cancers (6.1% of total diagnosed cases and 9.2% of total cancer deaths).^91^ YB‐1 can induce oxaliplatin resistance by interacting with NONO and RALY in colorectal cancer cells. Thus, knock‐down of NONO/RALY significantly sensitized YB‐1‐overexpressing colorectal cancer cells to oxaliplatin treatment.^103^ Additionally, GAPLINC binds to NONO/PSF, subsequently promoting cancer metastasis.^73^

As a protooncogene, Spi‐a/PU.1, an Ets‐related transcription factor, is usually overexpressed in Friend erythroleukaemia.^104^ Up‐regulated Spi‐1/PU.1 induces Friend erythroleukaemia via its interaction with NONO protein. Mechanistically, NONO binds Spi‐1/PU.1 via its RNA binding domain, and NONO splicing function is interfered by this binding.^33^ In human acute monocytic leukaemia THP1 cells, NONO is strongly expressed, while knock‐down of NONO slightly promotes cell proliferation but strongly inhibits motricity and invasion.

In primary neuroblastoma, high NONO expression level is correlated with N‐Myc expression; associated with poor patient prognoses; strongly associated with reduced overall survival; and independent of disease stage, age at the time of diagnosis and MYCN amplification.^79^ Differential proteomic analysis in bladder cancer demonstrated that NONO is strongly correlated with vascular invasions and appeared to be significantly (*P* < .0001) associated with a decreased probability of survival.^75^ Another computational genomic analysis demonstrated that NONO is significantly overexpressed in malignant pleural mesothelioma (MPM) and that NONO‐induced suppression of collagen biogenesis could be a nodal event in MPM.^105^

## 7 CONCLUSION AND OUTLOOK

We have summarized the evidence that NONO plays important roles in human tumorigenesis. Unlike most ‘normal’ tumour/anti‐tumour proteins, NONO has roles in tumorigenesis not only at the protein level but also at the DNA level. *NONO* is one of partner genes that has been identified as a fusion partner of *TFE3* in RCC. Whether *NONO* has other gene partners or whether other cancers can occur from gene fusions involving *NONO* is currently unresolved. Apparently, NONO has specific roles under different contexts. The literatures examined in our review support the notion that NONO is overexpressed in most cancers, induces/promotes cell proliferation, inhibits apoptosis, impairs DNA damage repair and has other roles promoting tumorigenesis. Meanwhile, there are some cancers in which NONO is down‐regulated, and these lower NONO levels also promote cancer progression. The best example of this relationship is breast cancer, in which the loss or change in NONO expression, along with the loss of ER, results in more aggressive forms of the disease. Another interesting area is the regulation of NONO at the transcriptional and/or translational level. Not all cell lines revealed a clear correlation between NONO mRNA and protein expression levels, demonstrating that NONO could be likely post‐transcriptionally regulated.^11^ Recently, two E3 ubiquitin ligases were proven to mediate NONO ubiquitination,^14^, ^28^ though whether there is a deubiquitylase for NONO is still unclear. NONO is primarily distributed within the nucleus, although it is also found in the cytoplasm, and it increases gradually as breast cancer progresses.^106^ However, understanding the particular roles of NONO DNA, RNA, and protein in various cell processes in detail will require more studies in the future. Better understanding of the context of NONO’s functions in cells and tumorigenesis will make it therapeutically invaluable.

## CONFLICT OF INTERESTS

The authors declare that no competing financial interests exist.

## AUTHOR CONTRIBUTIONS

Mao Ye and Lei Zhang involved in the conception and design of the study. Peifu Feng, Ling Li, Tanggang Deng, Yan Liu, Neng Ling, Siyuan Qiu, Lin Zhang, Bo Peng, Wei Xiong, Lanqin Cao collected and assembled the data. Peifu Feng, Mao Ye, Ling Li and Lei Zhang interpreted and analysed the data.: Peifu Feng and Mao Ye involved in the writing of the manuscript. Mao Ye and Lei Zhang contributed to the administrative support. All authors involved in the final approval of the manuscript.
